# Heart failure and oral bacteria: How could be prevented?

**DOI:** 10.4103/0975-3583.70924

**Published:** 2010

**Authors:** Ali M. Tavana

**Affiliations:** *Health Research Center, Baqiyatallah (a.s), University of Medical Sciences, Tehran, Iran*

Sir,

It has to be said that more than 400 bacterial species are localized in the subgingival plaque.[1,2] Very common bacteria species—*Actinobacillus actinomycetemcomitans, Fusobacterium nucleatum, Tannerella forsythensis (Bacteroides forsythus), Porphyromonas gingivalis, Prevotella intermedia*, and spirochetes have also been identified from symptomaticinfected implants.[3,4] Bacterial infection is the main reason of heart failure.[5] Nowadays different studies have shown that different bacteria are present in the pocket of patient with heart diseases,[6] different factors may have a role in implementation of heart diseases including oral infection[7] and systemic diseases.[8,9] It is supposed that specific oral organisms may have a role for heart failure.[10] Perhaps the role of many oral bacteria is also not clear in heart failure, and the mechanisms of this relationship are not completely known.[11] Possibly specific organism may be involved in different heart failure, but many more studies are necessary to clear the fact. Good oral hygiene could be more effective against heart diseases, in particular, in people who had a history of heart diseases among their family or previous heart attacks. It seems oral health is the key before and after heart diseases in patients, and it should not be forgotten by patients.
